# Post-Operative Outcome Predictions in Vestibular Schwannoma Using Machine Learning Algorithms

**DOI:** 10.3390/jpm14121170

**Published:** 2024-12-22

**Authors:** Abigail Dichter, Khushi Bhatt, Mohan Liu, Timothy Park, Hamid R. Djalilian, Mehdi Abouzari

**Affiliations:** Division of Neurotology and Skull Base Surgery, Department of Otolaryngology-Head and Neck Surgery, University of California, Irvine, CA 92697, USA

**Keywords:** vestibular schwannoma, artificial neural network, complication, machine learning, reoperation

## Abstract

**Background/Objectives:** This study aimed to develop a machine learning (ML) algorithm that can predict unplanned reoperations and surgical/medical complications after vestibular schwannoma (VS) surgery. **Methods:** All pre- and peri-operative variables available in the American College of Surgeons National Surgical Quality Improvement Program (ACS-NSQIP) database (n = 110), except those directly related to our outcome variables, were used as input variables. A deep neural network model consisting of seven layers was developed using the Keras open-source library, with a 70:30 breakdown for training and testing. The feature importance of input variables was measured to elucidate their relative permutation effect in the ML model. **Results:** Of the 1783 patients with VS undergoing surgery, unplanned reoperation, surgical complications, and medical complications were seen in 8.5%, 5.2%, and 6.2% of patients, respectively. The deep neural network model had area under the curve of receiver operating characteristics (ROC-AUC) of 0.6315 (reoperation), 0.7939 (medical complications), and 0.719 (surgical complications). Accuracy, specificity, and negative predictive values of the model for all outcome variables ranged from 82.1 to 96.6%, while positive predictive values and sensitivity ranged from 16.7 to 51.5%. Variables such as the length of stay post-operation until discharge, days from operation to discharge, and the total hospital length of stay had the highest permutation importance. **Conclusions:** We developed an effective ML algorithm predicting unplanned reoperation and surgical/medical complications post-VS surgery. This may offer physicians guidance into potential post-surgical outcomes to allow for personalized medical care plans for VS patients.

## 1. Introduction

Vestibular schwannomas (VSs) are benign tumors that account for over 90% of cranial nerve schwannomas and 6–8% of intracranial tumors [1,2,3]. Since surgical resections of VS present with major challenges, many studies have characterized the complications and reoperations following VS surgery [4,5,6,7]. While the safety of this procedure has significantly improved over the past years because of advancements in surgical techniques [8], risks remain and complications and reoperations following VS surgery continue to be a substantial burden on patients and the healthcare system. This has heightened the importance of improving post-VS surgery predictive capabilities to minimize surgical outcome risk.

Recent developments in machine learning (ML) and increases in available annotated medical data have allowed for the successful application of these technologies to many fields of medicine [9,10,11]. Artificial neural networks (ANNs) are one type of ML that outperform traditional statistical methods when manipulating large datasets with an inherent nonlinear distribution that is not well understood [12]. Since many clinical variables, such as risk factors or treatment outcomes, possess multivariate causes, ANN’s features make it a strong candidate for predictive analyses in a wide variety of medical disciplines. However, it has yet to be applied to predictions of post-surgical outcomes for VS surgery.

Given such information, the aim of this study was to develop an ANN algorithm, specifically a deep neural network (DNN), that can predict unplanned reoperation, surgical complications, and medical complications after VS surgery. The American College of Surgeons National Surgical Quality Improvement Program (ACS-NSQIP) database was used to train and validate the algorithm. Such technology could serve as a useful predictive tool for mitigating the effect of given problems on patients and healthcare infrastructure alike.

## 2. Materials and Methods

Reporting 30-day morbidity and mortality information for many surgical operations, the ACS-NSQIP database was retrospectively reviewed for data from the years 2007 to 2019. A total of 1783 patients with a diagnosis of VS undergoing surgery were identified using the ICD-9 and ICD-10 codes 225.1 and D33.3. Out of these patients, we selected those who specifically underwent head and neck surgery according to current procedural terminology (CPT) codes related to VS surgery (see Table 1).

Three primary outcomes of interest were predicted: the occurrence of an unplanned reoperation, surgical complications, and medical complications. For reoperations, we used the “RETURNOR” variable, which indicates an unplanned reoperation. Multiple variables, such as blood transfusions within 72 h, wound disruptions, the occurrences of superficial surgical site infections (SSIs), organ/space SSIs, and deep SSIs, were used to determine surgical complications. Finally, medical complications included occurrences of renal insufficiency, pneumonia, being on ventilation for more than 48 h, unplanned reintubation, myocardial infarction, urinary tract infections, deep vein thrombosis, pulmonary embolism, acute renal failure, cerebrovascular accidents with neurological deficits, septic shock, cardiac arrest requiring cardiopulmonary resuscitation, or sepsis.

A total of 110 preoperative, operative, and post-operative variables were selected as input variables. The data were acquired from three separate Excel datasets that contained data concerning medical complications, reoperative cases, and surgical complications. All three datasets were imported into Python and merged into a single dataset. The CASEID (a unique identifier specific to each data entry and unique in structure for each data source) was used to discriminate between the data sources after merging. During the merge, some datasets were found to have available data that other datasets lacked. To resolve this, we consistently prioritized the available data for our analyses.

To prepare for analysis, binary variables, like LOS_binary (length of stay) and ASA_binary (ASA), were label-encoded to simplify the classification process. Multi-class categorical variables were one-hot encoded to keep them interpretable. The main outcome variables—REOPERATION (reoperation), Surg_Comp (surgical complications), and Med_Comp (Medical complications)—were converted to binary indicators, where 1 represented “yes” and 0 represented “No”.

Exploratory Data Analysis (EDA) was conducted to examine the relationships between predictor variables and outcomes. Statistical measures and visualization techniques were used, guided by a custom analyze_x_against_y function that streamlined the analysis of relationships between predictors and outcomes. Categorical variables were dummy-coded to ensure compatibility with machine learning models. Correlation matrices were generated to assess associations between predictors and outcomes. Additionally, mean ratio analysis was used to calculate the proportion of positive cases for each predictor, which helped us identify those variables that possessed strong links to outcomes.

A screening process using area under the curve of receiver operating characteristics (ROC-AUC) scores was employed next to assess each predictor’s ability to distinguish between outcomes. Categorical variables were evaluated using decision tree classifiers, whereas numerical variables were evaluated with logistic regression. Only those variables that possessed an ROC-AUC score of 0.55 or higher were retained for modeling. For predictive modeling, an XGBoost classifier was implemented for each outcome.

The model used a refined set of predictors and a strong classification pipeline. Predictors possessed an ROC-AUC score above 0.55, which included categorical and numerical variables that were selected based on the EDA results. To address any imbalance in the outcome data, the scale_pos_weight parameter was adjusted to improve the model’s accuracy by balancing positive and negative cases in the target variable. Each outcome dataset was split into training (70%) and testing (30%) subsets using stratified sampling. Key hyperparameters (including n_estimators, max_depth, learning_rate, subsample, colsample_bytree, lambda, and alpha) were fine-tuned using the GridSearchCV class to maximize the ROC-AUC score. Additionally, three-fold cross-validation within GridSearchCV improved model reliability by reducing overfitting.

Feature engineering was applied to procedural and work relative value unit (WRVU) codes. Procedural codes (CONCPT) were transformed into a binary matrix, which indicated the presence of each unique code for each case. An aggregated column was added to the matrix to represent the total number of procedures. WRVU codes were processed in a similar way, with counts capturing the extent of interventions each patient received. Each final model incorporated the original variables (e.g., CONCPT, CONWRVU, OTHERCPT, OTHERPROC, OTHERWRVU) to improve predictive accuracy.

Model performance was evaluated using accuracy, sensitivity, specificity, F1 score, ROC-AUC, precision–recall area under the curve (PR-AUC), negative predictive value (NPV), and positive predictive value (PPV). Receiver operating characteristics (ROCs) and precision–recall (PR) curves were plotted to visualize performance, and area under the curve (AUC) values were calculated for both. Feature importance was derived from the final models, and the top 20 predictors were visualized. The best-performing model for each outcome, along with the feature sets used, were saved in an organized directory to ensure reproducibility. The top 10 predictors for each outcome were extracted.

## 3. Results

This study comprised 1783 patients overall. In total, 765 (42.9%) were male patients and 1018 were female (57.1%). Furthermore, 1289 patients were white (72.3%), 83 (4.66%) were Black or African American, 82 (4.60%) were Asian, and 43 (2.41%) were Hispanic. The mean age of the cohort was 50.4 ± 13.9 years.

**Figure 1:**
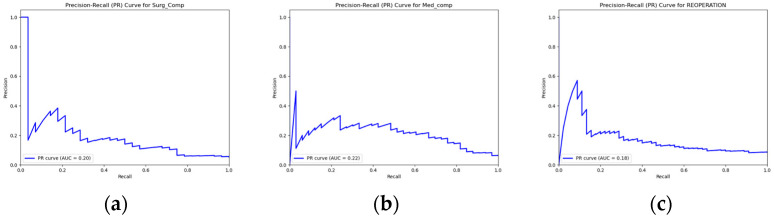
PR curves for predictions of (**a**) surgical complications; (**b**) medical complications; and (**c**) reoperation.

**Figure 2:**
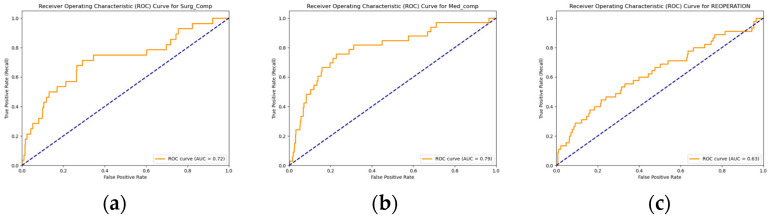
ROC curve for predictions of (**a**) surgical complications; (**b**) medical complications; and (**c**) reoperation.

Out of the total of 1783 patients, 151 (8.5%) had undergone a reoperation, 111 (6.2%) had experienced medical complications, and 92 (5.2%) had experienced surgical complications. The ROC-AUC value for the predicted occurrence of reoperation in the test dataset was 0.6315. The corresponding accuracy of prediction was 0.8206, the sensitivity was 0.3111, the specificity was 0.8673, the PPV (precision) was 0.1772, the NPV was 0.932, the F1 score was 0.2258, and the PR-AUC was 0.1968. The ROC-AUC score for the predicted occurrence of medical complications in the test dataset was 0.7939, along with an accuracy of 0.8692, a sensitivity of 0.5152, a specificity of 0.8924, a PPV of 0.2394, an NPV of 0.9655, an F1 score of 0.3269, and a PR-AUC of 0.2208. Finally, the ROC-AUC value for the predicted occurrence of surgical complications in the test dataset was 0.719, while the accuracy of prediction was 0.8729, the sensitivity was 0.3571, the specificity was 0.9014, the PPV was 0.1667, the NPV was 0.9621, the F1 score was 0.2273, and the PR-AUC was 0.1795. Table 2 and Figure 1 and Figure 2 relay the evaluation statistics, PR curves, and ROC curves for all predictions.

Finally, the permutation importance of each variable’s outcome-predicting capabilities was calculated. The top 10 most important features for predicting each outcome are listed in Table 3. Though there was some variance depending on the outcome variables, features including the length of stay post-operation until discharge, days from operation to discharge, and the total hospital length of stay were categorized as the most important predictive features.

## 4. Discussion

Reoperations and complications post-surgery undoubtedly place mental and physical tolls on patients [13]. It also places a burden on the healthcare system, given that rehospitalization post-discharge has been estimated to cost USD 17 billion annually in avoidable Medicare expenditures [14]. The Agency for Healthcare Research and Quality has been implemented to combat this issue; however, this has contributed to controversies regarding readmission rates and reimbursements [15]. A high-quality predictive model could mitigate this issue by offering physicians insights into potential post-surgical outcomes and allowing them to devise a more personalized treatment plan. ANN developments have enabled many researchers to apply this technology for predictions of various surgical variables including length of stay, readmission, complication, recurrence, and reoperation among various specialties [10,16,17]. However, similar studies in the literature regarding VS remain very scarce, and to our understanding, our study is one of the first to predict occurrences of reoperation, surgical complications, and medical complications following surgical removal of VS.

Our DNN algorithm reached an AUC-ROC of 0.6315, 0.7939, and 0.719 for predicting occurrences of reoperation, medical complications, and surgical complications, respectively. These results match those of other studies that utilized ANN algorithms to predict post-operation factors for other surgical procedures such as those in orthopedic surgery and neurosurgery [16,17]. Medical outcomes are dependent on a variety of factors, of which many of these are often based on complex relationships that may not be explicit. ANN is poised for these scenarios, since no previous knowledge regarding the importance of specific input variables is needed, and the model can also detect relationships between input and output variables automatically [18,19]. In contrast, other ML techniques, such as logistic regressions and random forest classifiers, perform well when the input dataset is relatively streamlined to include minimal variables that are known to be important in predicting the outcome variable [18]. Though it is oftentimes difficult to assess which types of algorithms are best suited for each task at hand, our results clearly show that a DNN algorithm was able to predict post-surgical VS complications and reoperations in the present study.

An interesting and meaningful aspect of our study was the characterization of variables that highly influenced the algorithm’s ability to predict occurrences of complications and reoperations. These variables could be monitored for future treatments of VS to potentially improve patient outcomes. Variable importance for ANN classifications cannot be currently measured directly, and hence, we utilized a method that measures model performance when a specific variable is removed from the dataset. Variables with a higher decrease in performance were regarded as more important. It was therefore notable that the length of stay post-operation until discharge, days from operation to discharge, and the total hospital length of stay were determined as the most important variables for predictions. Though this method provides insights into the importance of individual variables, it is important to note that it does not provide an explanation of how each variable precisely influences the prediction. ML explainability and interpretation is a topic that is under extensive investigation, and there is currently no method that extracts the true importance of each variable [20]. Hence, these findings should be referenced with caution.

While the negative predictive value of our model was very high, averaging at 0.953 between the three variables at a classification threshold of 0.55, the positive predictive value was relatively low, averaging at 0.194. This indicates that our DNN model was particularly proficient in identifying cases that were not followed by post-operative complications or reoperations and that positive cases were more difficult to predict given the variables inputted into the model. Patients with head and neck cancer have been identified as a high-risk group for readmission, with their rates ranging between 6% and 26.5% [21,22,23,24]. Though the Hospital Readmissions Reduction Program (HRRP) has not been implemented for head and neck surgery, many studies have pointed out the likeliness of the program expanding to cover such procedures in the future [25,26]. Our study has illustrated that a significant part of complication or reoperation negative VS cases were highly predictable. Hence, a predictive model based on the ANN algorithm developed through this study may become useful in the future for developing a stratified model in which hospitals will be penalized for post-surgical incidences only for cases that were predicted to have a low probability of such events occurring.

Similar predictive indices have been considered before, such as the LACE+ (length of stay, acuity, comorbidities, and emergency presentations) index, which predicts an early death or urgent readmission after hospital discharge [27]. Furthermore, the University of Kansas Health Systems and Atrium Health have both successfully implemented predictive programs that analyze clinical and socioeconomic factors after hospital discharge, resulting in a successful reduction in their readmission rates [28]. Nonetheless, neither program has employed ML techniques. Incorporating the predictive capacity of ANN or alternate ML algorithms into such frameworks could improve the quality of these risk-stratification models, allowing healthcare professionals to appropriately measure the risk of post-surgical outcomes, thereby benefiting not only the healthcare system but also patients by providing them with more information and tailored guidance regarding their treatment.

Despite the many findings, it is important to mention several limitations of our study. The performance of classifiers is inherently rooted in the quality of the training dataset, which, in the case of this present study, was the ACS-NSQIP database. Most datasets are subject to errors, and this is no different for the NSQIP database, as shown by a previous study that reported inaccuracies in CPT coding among neurosurgical procedures [29]. This database also may not be generalizable to all intended patients, given that the database only included a maximum of 708 hospitals [30]. Furthermore, the input variables of our model are limited to those included in the database. This inevitably omits potentially relevant variables for predicting post-surgical VS incidents, such as the presence of NF2 mutation status, tumor size or location, or surgical approaches, which could all impact the outcome of treatment [31,32].

A class imbalance also existed between cases of patients who presented without any post-surgical reoperations or complications and those who did, with all percentages being below 8.5%. Future adjustments, such as increasing the size of the dataset, may improve positive case detection and balance. On a similar note, the NSQIP database presented a plethora of missing data. Though we maneuvered this challenge by prioritizing the available data, in future studies, a more complete dataset could improve the performance of the algorithm.

## 5. Conclusions

A robust ML algorithm predicting unplanned reoperation and surgical or medical complications following VS surgery was developed. The model possessed an ROC-AUC of 0.6315, 0.7939, and 0.719 for prediction reoperation, medical complications, and surgical complications, respectively. Variables deemed to be most important for making predictions included those such as the length of stay post-operation until discharge, days from operation to discharge, and the total hospital length of stay. The above findings may offer physicians insights into potential post-surgical outcomes, though further investigations incorporating new algorithms and databases may be needed for the clinical implementation of a more personalized treatment plan for VS patients.

## Figures and Tables

**Table 1:** List of procedures and associated CPT codes related to VS surgery. CTBC, Craniectomy, Trephination, and Bone Flap Craniotomy; IPF, Infratentorial or Posterior Fossa; CBTC = Craniectomy, Bone Flap Craniotomy, and Transtemporal Excision; PJM, Posterior Cranial Fossa, Jugular Foramen, or Midline Skull Base; IPP, Infratemporal Fossa, Parapharyngeal Space, or Petrous Apex; PCF, Posterior Cranial Fossa, Clivus, or Foramen Magnum.

Procedure	CPT Code
Supratentorial Craniectomy or Craniotomy Exploratory	61304
Craniectomy or Craniotomy, Exploratory, Infratentorial	61305
Suboccipital Craniectomy with Cervical Laminectomy	61343
Posterior Fossa Cranial Decompression	61345
Suboccipital Craniectomy	61458
Suboccipital Craniectomy—Section of Cranial Nerves	61460
Craniectomy with Tumor or Bone Lesion Excision	61500
CTBC for Supratentorial Tumor	61510
CTBC for Supratentorial Meningioma	61512
CTBC for Supratentorial Cyst	61516
Craniectomy for IPF Brain Tumor	61518
Craniectomy for IPF Meningioma Brain Tumor	61519
IPF Brain Tumor Excision or Cerebellopontine Angle Tumor Excision	61520
Excision of Midline Tumor at IPF Skull Base	61521
Brain Abscess Excision via IPF Craniectomy	61522
IPF Cyst Excision	61524
CBTC of Cerebellopontine Angle Tumor	61526
CBTC of Cerebellopontine Angle Tumor with Posterior Fossa Craniotomy	61530
Craniotomy with Partial or Subtotal Hemispherectomy	61543
Craniotomy for Pituitary Tumor Removal with Intracranial Approach	61546
Pituitary Tumor Excision via Transnasal or Transseptal Approach	61548
Craniectomy/Craniotomy with Foreign Body Removal	61570
Craniofacial Approach to Anterior Cranial Fossa	61581
Infratemporal Pre-Auricular Approach to Middle Cranial Fossa	61590
Infratemporal Post-Auricular Approach to Middle Cranial fossa	61591
Orbitocranial Zygomatic Approach to Middle Cranial Fossa	61592
Transtemporal Approach to PJM	61595
Transcochlear Approach to PJM	61596
Transcondylar Approach to PJM	61597
Transpetrosal Approach to PCF	61598
Lesion Reduction in IPP, specifically Extradural Area	61605
Resection of Lesions in IPP	61606
Resection of Lesions in Parasellar Area, Cavernous Sinus, Clivus, or Midline Skull Base	61608
Resection of Lesions at PCF	61615 and 61616
Secondary Repair of Dura Post-Skull Base Surgery	61618
Craniectomy or Craniotomy for Neurostimulator Electrode Implantation on Cerebral Cortex	61860
Dural or CSF Leak Repair	62100
Lumbar Intraspinal Lesion Removal via Laminectomy	63267
Extradural Growth of Spinal Cord via Laminectomy	63277
Laminectomy with Tethered Spinal Cord Release in Lumbar Region	63200
Intradural, Extramedullary Growth of Spinal Cord via Laminectomy	63281
Excision of Intradural, Extramedullary Growth on Lumbar Spinal Cord	63282
Intradural, Intramedullary Growth in Cervical Spine via Laminectomy	63285
Excision of Intradural, Intramedullary Neoplasm via Laminectomy in Thoracolumbar Region	63287

**Table 2:** Model performance statistics for predicting the occurrence of reoperation, medical complications, and surgical complications (classification threshold = 0.55).

Metric	Reoperation	Medical Complications	Surgical Complications
Accuracy	0.8206	0.8692	0.8729
Sensitivity	0.3111	0.5152	0.3571
Specificity	0.8673	0.8924	0.9014
Precision	0.1772	0.2394	0.1667
F1 Score	0.2258	0.3269	0.2273
ROC-AUC	0.6315	0.7939	0.719
PR AUC	0.1968	0.2208	0.1795
NPV	0.932	0.9655	0.9621
PPV	0.1772	0.2394	0.1667

**Table 3:** The top 10 important variables for predictions of a reoperation occurrence, medical complication, and surgical complication. ALKPHOS, alkaline phosphatase; WBC, white blood count; INR, international normalized ratio; SGOT, Serum Glutamic Oxaloacetic Transaminase; ASA, American Society of Anesthesiologists.

Ranking	Reoperation	Medical Complication	Surgical Complication
1	Days from Operation to Discharge	Hospital Discharge Destination Other than Home	Length of Stay Post-Operation until Discharge
2	Total Hospital Length of Stay	Total Hospital Length of Stay	Triage Operation Time
3	Time Duration from ALKPHOS Preoperative Labs to Operation	Days from Operation to Discharge	Days from Hospital Admission to Operation
4	Time Duration from WBC Preoperative Labs to Operation	Hypertension Requiring Medication	Total Hospital Length of Stay
5	Time Duration from INR Preoperative Labs to Operation	Time Duration from INR Preoperative Labs to Operation	Total Operation Time
6	Preoperative SGOT	Preoperative Serum Albumin	Preoperative Total Bilirubin
7	Total Operation Time	Total Operation Time	Days from Operation to Discharge
8	Time Duration from Bilirubin Preoperative Labs to Operation	Time Duration from Platelet Count Preoperative Labs to Operation	Preoperative SGOT
9	Age of Patient	ASA Classification	Time Duration from WBC Preoperative Labs to Operation
10	Triage Operation Time	Age of Patient	ASA Classification

## Data Availability

The data that support the findings of this study are available from the corresponding authors upon reasonable request.
